# Socioeconomic Position and Health-Seeking Behavior for Hearing Loss Among Older Adults in England

**DOI:** 10.1093/geronb/gbu024

**Published:** 2014-03-24

**Authors:** Lenka Benova, Emily Grundy, George B. Ploubidis

**Affiliations:** ^1^Department of Population Health, London School of Hygiene and Tropical Medicine, London, UK.; ^2^Department of Social Policy, The London School of Economics and Political Science, London, UK.

**Keywords:** Health-seeking behavior, Hearing loss, Socioeconomic gradient.

## Abstract

**Objective.:**

To examine whether socioeconomic position (SEP) is associated with progression in the health-seeking process for hearing loss.

**Method.:**

Logistic regression of data from a cross-sectional survey representative of noninstitutionalized, 50 years and older population of England (ELSA wave 2, 2004). Using self-reported hearing difficulty as starting point, we examined the association between SEP and health-seeking behaviors in 6 stages leading to hearing aid acquisition and use.

**Results.:**

Higher SEP was associated with lower odds of self-reported hearing difficulty, adjusted odds ratio [OR] = 0.87 (95% confidence interval [CI] 0.83–0.91, *p* < .001). There was marginal negative association between higher SEP and receiving hearing aid recommendation (adjusted OR = 0.88, 95% CI 0.78–0.99, *p* = .05). SEP was not associated with any other stage of health-seeking behavior.

**Discussion.:**

Among the noninstitutionalized older population of England, SEP-related inequalities exist in the prevalence of self-reported hearing loss. However, SEP is not strongly associated with progression in the remaining stages of health-seeking process during and after an individual’s contact with the health system.

Hearing loss is predicted to become the seventh most important contributing factor to the burden of disease by 2030 worldwide (World Health Organization, 2008). The recent Global Burden of Disease 2010 estimates showed that hearing loss ranked as the 17th leading factor of years lived with disability in the United Kingdom, highest of all sensory impairments (Murray et al., 2013). A 2011 study found that 45% of men and 39% of women older than 60 years in the United Kingdom suffered from moderate or worse hearing impairment, defined as >35 hearing level in decibels (dB HL) in the better ear averaged over 0.5, 1, 2, and 4kHz frequencies (Davies, 2012). The second wave of the English Longitudinal Study of Ageing (ELSA) in 2004 reported prevalence of self-reported hearing difficulty among individuals aged 65 and older at 45% (Banks, Breeze, Lessof, & Nazroo, 2006).

Hearing impairment occurs among older adults as a result of the aging process but can be exacerbated by additional factors such as noise exposure, hereditary conditions, health conditions, and medical treatments (Alpiner & Roche, 1996; Ruan, Ma, Zhang, & Yu, 2013). This hearing impairment (presbycusis) most often presents as a combination of decreased ability to hear higher frequencies and at lower volumes, leading to reduced speech recognition, especially in environments with background noise. The onset of presbycusis is gradual, with a mean reported duration at diagnosis of 10 years (Davis, Smith, Ferguson, Stephens, & Gianopoulos, 2007). It is therefore not easily apparent to the affected individual, who may compensate via verbal, nonverbal, and maladaptive (avoidance) communication strategies (Helvik, Wennberg, Jacobsen, & Hallberg, 2008). The prevalence of hearing impairment is higher among adult men than women (Stevens et al., 2011) and is associated with other health comorbidities, such as poor nutrition and smoking (Heine, Browning, Cowlishaw, & Kendig, 2013; Yamasoba et al., 2013).

Studies from high-income countries show that hearing impairment among older people is underdiagnosed and undertreated. In Australia, 36% of those who failed a telephone screening test sought help, and only 4% obtained hearing aids (Meyer et al., 2011). Among people older than 80 years in Finland, 75% of those with moderate hearing impairment were not using hearing aids (Hietanen, Era, Sorri, & Heikkinen, 2004). In the United Kingdom, less than half of older adults (60+ years) with severe hearing impairment (>75 dB HL) use hearing aids (Davies, 2012).

Among older adults, untreated hearing impairment is associated with deterioration in physical, mental, and psychosocial functioning, as well as with mortality (Appollonio, Carabellese, Frattola, & Trabucchi, 1996; Dalton et al., 2003; Hietanen et al., 2004; Kramer, Kapteyn, Kuik, & Deeg, 2002; Lin et al., 2013). Hearing aid use was associated with improved communication ability (Yueh et al., 2010), better quality of life (Mondelli & Souza, 2012), higher mental health scores (Gopinath, Schneider, Hickson, et al., 2012), and less loneliness (Pronk et al., 2011). On the other hand, nonuse of assisted listening devices was shown to reduce quality of life of the affected individual (Dalton et al., 2003; Gopinath, Schneider, McMahon, et al., 2012) and also carries negative implications for their social environment (Wallhagen, Strawbridge, Shema, & Kaplan, 2004).

Differences in health outcomes based on social and economic background (SEP gradients) are recognized as avoidable and unfair inequalities that must be interrogated and addressed (Marmot, 2005). Health-seeking behavior is one of the direct pathways through which socioeconomic status can influence health outcomes (Stowasser, Heiss, McFadden, & Winter, 2011). Health-related behavior was cited alongside educational opportunities, income distribution, and access to health care as an important area where intervention may improve health outcomes (Mackenbach et al., 2008). Increased life expectancy, population aging, and the concentration of ill-health among the older age group in high-income countries have highlighted the importance of examining pathways leading to socioeconomic inequalities in later life health.

A formal comparison of causal mechanisms linking socioeconomic position (SEP) and health outcomes among the older population in England showed that financial resources and behaviors, rather than psychosocial factors, were significantly associated with gradients in health status (Ploubidis, DeStavola, & Grundy, 2011). Therefore, the cost of care and use of information in accessing health care, both critical components of health-seeking behaviors, could play a role in determining the existence and extent of a potential SEP gradient in hearing loss treatment in England. Availability of free universal care may not completely eliminate the socioeconomic gradient in health-seeking behavior. Studies have shown that among older adults in the United Kingdom, individual SEP was associated with health-seeking behaviors, such as uptake of screening for cancer (Maheswaran, Pearson, Jordan, & Black, 2006; Miles, Rainbow, & Von Wagner, 2011) and receiving influenza vaccination (Patel, Lawlor, & Ebrahim, 2007). Although direct costs of care may be minimal, indirect costs of, for instance, transportation and time trade-off may be important, as can other characteristics of the interaction between the individual and the health service (quality of care, trust, and communication effectiveness). Additionally, a complex interaction of factors, such as acceptance of the condition, perception of stigma, attitudes of relatives, expectations of benefit from hearing aid, and hearing aid comfort levels, may be socially patterned (Saunders, Chisolm, & Wallhagen, 2012).

Current research about socioeconomic determinants of health seeking for hearing loss is limited mainly to examination of hearing aid ownership. A recent systematic literature review did not identify any studies examining the association between SEP and likelihood of reporting hearing difficulty to a health professional (Knudsen, Öberg, Nielsen, Naylor, & Kramer, 2010). Among older individuals in the United States, greater income was associated with higher satisfaction, uptake, and use of hearing aids (Garstecki & Erler, 1998), and college education was linked with increased hearing aid acquisition (Fischer et al., 2011). Among a sample of Finnish hearing aid owners older than 75 years, lower income was associated with nonuse (Lupsakko, Kautiainen, & Sulkava, 2005). Stigma associated with hearing loss may also partly contribute to low hearing aid use (Gopinath et al., 2011; Wallhagen, 2010). A study among older people in London found that reluctance to seek health care may arise from low expectations and resignation to the health condition itself or to the fact that available treatment options are insufficient (Walters, Iliffe, & Orrell, 2001).

## Objective

**Figure 1.**
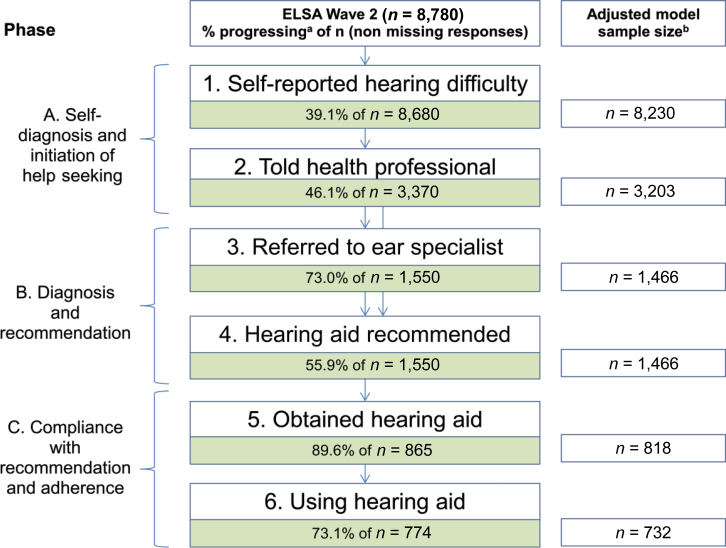
Proportions of ELSA Wave 2 sample progressing through six stages of health-seeking behavior for self-reported hearing difficulty. ^a^Complex survey design was accounted for in calculations of proportions. ^b^Sample size in adjusted analysis excluded observations with missing data in health-seeking behavior, socioeconomic position, gender, age group, marital status, retirement status, or ownership of private health insurance.

Untreated hearing difficulty can negatively impact on mental and physical health, yet hearing impairment among older individuals is underdiagnosed and undertreated in the United Kingdom (Smeeth et al., 2002). We were unable to identify any published studies linking SEP with health-seeking behavior related to hearing loss in the United Kingdom. Therefore, in this article, we present an exploratory analysis of the progression from self-reported hearing difficulty to acquiring and using a hearing aid in order to elucidate whether health-seeking behavior for hearing difficulty among older people in England is socioeconomically patterned. In order to guide policy decisions and allocation of resources, we examine whether any such differentials originate before, during, or after a person’s interaction with the health care system, corresponding to three broad phases (Figure 1). The fact that there is currently no hearing loss screening program among older people in the United Kingdom (Mackie, 2009) enables the analysis of health-seeking behavior to start with self-diagnosis and the individual’s initiation of contact with a health provider (Phase A: Stages 1 and 2). Phase B entails progression through stages 3 and 4—being referred to an ear specialist and receiving a recommendation for a hearing aid—both of which take place in the health care system. Lastly, individuals evaluate their needs, advice received during their clinical encounters, as well as potential benefits of adhering to such advice, before deciding whether to obtain and use a hearing aid (Phase C: Stages 5 and 6).

## Materials and Methods

### The Sample

This study used data from the second wave of the ELSA due to availability of self-reported information on the existence of hearing difficulty and consecutive multiple stages of health-seeking behavior. ELSA core members are a representative sample of the noninstitutionalized population who were 50 years or older on March 1, 2002, and living in England. The ELSA cohort originated from the Heath Survey for England (HSE or ELSA Wave 0), which provided enrollment of more than 12,000 core members and baseline data about them between years 1998 and 2001 (Cheshire, Cox, Lessof, & Taylor, 2006; Taylor, Conway, Calderwood, & Lessof, 2002). The sample for this study is based on the 8,780 core members for whom data were collected in ELSA Wave 2 in 2004/2005. Cross-sectional analysis of association between SEP and progression in health-seeking behaviors for hearing difficulty was conducted on this sample. Sample weights provided with the data set were used to adjust for nonresponse between HSE and ELSA Wave 1 and between ELSA wave 1 and 2 (82% of Wave 1 core members participated in Wave 2) in order to retain the representativeness of the sample.

### Outcome Measures: Progression in Stages of Health-Seeking Behavior

Respondents were asked to rate their hearing on a scale from excellent, very good, good, fair to poor. In addition, they were asked whether they had difficulties following a conversation in a setting with background noise. If respondents rated their hearing as fair or poor, or acknowledged having difficulties following a conversation, they were asked whether they had mentioned their hearing problems to a health professional (physician or nurse). An affirmative response led to being asked whether the respondents were referred to an ear specialist. All individuals who mentioned their hearing difficulty to a health professional were asked whether they received a recommendation for a hearing aid and if so, whether they obtained one. The section concluded by asking whether the respondent uses their hearing aid. These six questions were considered discrete stages in the health-seeking process, and binary responses to them were analyzed separately.

All stages of health-seeking behavior, starting with perception of hearing difficulty, were self-reported and not confirmed by other methods. The reliability of the self-reported measure of hearing loss among older people compared to audiometry ranges between populations, depends on severity of hearing impairment, and may vary by demographic characteristics. In Taiwan, 21.4% of older people with moderate to profound hearing impairment (>40 dB HL as measured by audiometry) self-reported hearing difficulty (Chang, Ho, & Chou, 2009). Among older adults with high prevalence of hearing impairment in Finland and Australia, a single self-report question was found by authors to be sufficiently sensitive to detect moderate or worse hearing impairment and thus suitable for prevalence surveys if audiometry is not available (Salonen, Johansson, Karjalainen, Vahlberg, & Isoaho, 2011; Sindhusake et al., 2001). A systematic review of performance of single question compared to audiometry among the older population that identified 10 longitudinal studies concluded that a single question showed sufficiently good performance to be recommended for studies of hearing loss up to 2 or 4kHz at 40 dB HL in the better ear (Valete-Rosalino & Rozenfeld, 2005). Self-reported hearing difficulty may overestimate impairment at <70 years but underestimate it at >70 years (Kiely, Gopinath, Mitchell, Browning, & Anstey, 2012). It is not the aim of this study to estimate the prevalence of hearing impairment in the older population in England but to examine SEP gradients in the health-seeking process resulting from self-perceived hearing difficulty. Self-reported measure of hearing handicap can be a useful tool to identify individuals who require further screening and referral (Bagai, Thavendiranathan, & Detsky, 2006). We were unable to confirm the existence or the severity of hearing impairment among individuals who reported hearing difficulty. However, if such individuals progressed through the stages of health-seeking behavior to a hearing aid recommendation by a health professional or ear specialist (stage 4), the existence of hearing impairment was likely verified.

### Exposure: SEP

Education, occupation, income, and wealth were selected as the four indicators capturing most of the variation in socioeconomic situation (Galobardes, Shaw, Lawlor, Lynch, & Davey Smith, 2006) and used to construct a latent variable signifying SEP based on an earlier analysis of the ELSA sample (Ploubidis et al., 2011). Net income and net financial nonhousing wealth quintiles were used to capture relative financial position. Five categories of most recent self-reported occupation were used, managerial and professional; intermediate; small employees and own account workers; lower supervisory and technical workers; and workers in semiroutine occupations. Lastly, educational status was considered in five groups. First group included respondents with degree or equivalent qualifications, the second participants with GCE A level or equivalent qualifications, the third those with O levels CSE qualifications or equivalent, the fourth individuals with foreign qualifications, and the fifth category comprised those without a formal educational qualification. The latent variable was constructed so that a higher score signified higher socioeconomic standing, ranging from a minimum of −0.837 to maximum of 3.220. The standardized factor loadings of each of the four indicator variables were satisfactory, ranging from 0.50 to 0.76.

### Other Variables

Gender and age (three age groups, 50–64, 65–74, and 75+) were used in the analysis, due to their association with prevalence and extent of hearing impairment (Smeeth et al., 2002). In addition, current binary marital status was expected to be associated both with SEP and with progression through the various stages of health-seeking behavior for hearing difficulty (Chang et al., 2009; Duijvestijn et al., 2003; Pandhi, Schumacher, Barnett, & Smith, 2011). Lastly, binary retirement status and self-reported ownership of private health insurance were assessed for possible confounding in the association between SEP and health-seeking behavior (Braunack-Mayer & Avery, 2009).

### Statistical Analysis

Data were analyzed using Stata/SE v.12 (Stata Corporation, College Station, Texas). At each of the six stages of health-seeking behavior, a single model was estimated incorporating the continuous latent measure of SEP and adjusted for gender, age group, current marital status, retirement status, and ownership of private health insurance. Based on previous findings of the importance of gender and cohort effects in the pathways linking socioeconomic status with health outcomes this population (Ploubidis, Benova, Grundy, Laydon, DeStavola, 2014), gender, age group, and SEP were considered as potential effect modifiers and assessed in all six final adjusted models using the likelihood ratio (LR) test. If the LR test *p* value equalled <.05 in any of the six models, the modification effect was examined and reported.

Prevalence of self-reported hearing difficulty was estimated taking the sampling weights and design into account using the Stata “svy” command. Bootstrapping with 1,000 replications was used in each model, and the results of final adjusted models were confirmed using logistic regression including sample weights. The largest percentage of records with missing data in any of the variables was recorded at the first stage (6.3%), and due to low proportion of missingness (<5.7%) in the consecutive stages, records with missing data were eliminated from analysis.

## Results

Men comprised 45% of the initial sample of 8,780 (Table 1). Nearly 58% of the sample had held intermediate or higher occupations, and 60.5% completed secondary or higher educational qualifications. The mean SEP score among the 8,399 individuals in the sample for whom this measure was available reached 1.12 with a standard deviation of 1.03. The overall prevalence of self-reported hearing difficulty was 39.1% (95% confidence interval [CI] 38.0–40.2), increased by age group and was higher among men compared with women in every age group. Among individuals reporting hearing difficulties, 46.1% had mentioned the issue to a health professional. Within this group, 73.0% were referred to an ear specialist and 55.9% were recommended a hearing aid. From the individuals who received a recommendation for a hearing aid, 89.6% reported obtaining one. Among these hearing aid owners, 73.1% reported using the device (Figure 1). Overall, 15.9% of men and 17.9% of women with self-reported hearing difficulty used a hearing aid.

**Table 1.** Demographic, Health, and Socioeconomic Characteristics of the Sample

	Men	Women	Total
*n*	3,949	4,831	8,780
	Column %	Column %	Column %
Age group			
50–64	47.2	45.7	46.4
65–74	31.0	28.6	29.7
75+	21.8	25.6	23.9
Currently married			
No	24.6	42.9	34.7
Yes	75.4	57.1	65.3
Missing	0.0	<0.1	<0.1
Retirement status			
Not retired	43.2	47.1	45.3
Retired	55.7	52.4	53.9
Missing	1.2	0.6	0.8
Owns private health insurance			
No	84.6	86.3	85.5
Yes	15.4	13.6	14.4
Missing	<0.1	<0.1	<0.1
Occupation			
Managerial and professional	35.3	21.2	27.5
Intermediate	8.9	24.5	17.5
Small employees and own account workers	13.4	6.8	9.8
Lower supervisory and technical workers	16.2	6.7	11.0
Workers in semiroutine occupations	23.8	37.2	31.2
Missing	2.4	3.6	3.0
Education			
Degree/Higher education	30.1	18.7	23.8
A level	8.0	5.3	6.6
O level/CSE grade	23.1	19.9	21.3
Foreign/Other	5.2	11.6	8.7
No qualifications	33.5	44.4	39.5
Missing	0.1	0.1	0.1
Net financial wealth			
First quintile (highest)	18.4	20.9	19.7
Second quintile	17.8	21.3	19.7
Third quintile	19.9	19.6	19.7
Fourth quintile	21.1	18.6	19.7
Fifth quintile (lowest)	21.4	18.1	19.7
Missing	1.4	1.5	1.5
Income			
First quintile (highest)	16.2	22.6	19.7
Second quintile	19.0	20.4	19.7
Third quintile	19.9	19.6	19.7
Fourth quintile	21.1	18.4	19.7
Fifth quintile (lowest)	22.4	17.5	19.7
Missing	1.4	1.5	1.5
SEP			
*n*	3,792	4,607	8,399
Mean	1.32	0.99	1.12
Standard deviation	1.02	1.01	1.03

*Note.* SEP = socioeconomic position. Complex survey design was accounted for in calculations of proportions.

### Phase A: Prevalence of Self-Reported Hearing Difficulty and Initial Help Seeking

In the adjusted model, self-reported hearing difficulty was strongly associated both with gender and with age group (Table 2, model 1). There was a strong association between SEP and self-report of hearing difficulty, where an increase in one unit of SEP score was associated with a 13% decrease in the odds of self-reported hearing difficulty. There was some evidence of interaction between SEP score and age group, which was further examined in Table 3. The model including an interaction between SEP and age group showed strong evidence of an association between higher SEP score and lower odds of self-reported hearing loss in the two younger age groups, but not in the 75 and older age group. In the second stage, we did not observe an association between SEP and the odds of reporting hearing difficulty to a health professional among individuals reporting hearing difficulty (model 2). However, women had 19% lower and people in the oldest age group nearly double the odds of approaching a health professional about their hearing difficulty.

**Table 2.** Adjusted Models Showing Association Between Socioeconomic Position (SEP) and Health-Seeking Behavior Related to Hearing Loss in Six Stages of Health-Seeking Process

	OR	95% CI	OR	95% CI
Phase A	1. Self-reported hearing difficulty^a^, *n* = 8,230		2. Told health professional, *n* = 3,203	
SEP	0.87***	0.83–0.91	0.99	0.92–1.07
Gender				
Female	0.54***	0.50–0.60	0.81**	0.70–0.93
Age group				
50–64	Ref		Ref	
65–74	1.31***	1.15–1.49	1.19	0.98–1.46
75+	2.25***	1.95–2.59	1.89***	1.53–2.33
LR test	*χ* ^2^ = 33.05	*p* < .001	*χ* ^2^ = 0.04	*p* = .842
Phase B	3. Referral to ear specialist, *n* = 1,466		4. Hearing aid recommendation, *n* = 1,466	
SEP	0.93	0.82–1.06	0.88*	0.78–0.99
Gender				
Female	1.00	0.79–1.28	1.00	0.80–1.27
Age group				
50–64	Ref		Ref	
65–74	1.35	0.95–1.92	1.83***	1.32–2.54
75+	2.05***	1.43–2.93	5.11***	3.64–7.16
LR test	*χ* ^2^ = 1.14	*p* = .285	*χ* ^2^ = 4.13	*p* = .042
Phase C	5. Hearing aid obtained^a^, *n* = 818		6. Hearing aid use, *n* = 732	
SEP	1.25	0.97–1.60	1.09	0.91–1.32
Gender				
Female	1.61	0.95–2.75	1.20	0.82–1.77
Age group				
50–64	Ref		Ref	
65–74	3.02**	1.45–6.26	1.13	0.65–1.98
75+	4.21***	2.20–8.08	1.64	0.96–2.79
LR test	*χ* ^2^ = 2.85	*p* = .092	*χ* ^2^ = 0.90	*p* = .343

*Notes*. CI = confidence interval; LR = likelihood ratio; OR = odds ratio. LR test *χ* ^2^ and *p* value of the model comparing adjusted model with SEP to adjusted model without SEP.

All models adjusted for gender, age group, marital status, retirement status, and ownership of private health insurance.

^a^Interaction effect identified and further described in Table 3.

*p* value for Wald test, levels of significance: *p < .05. **p < .01. ***p < .001.

**Table 3.** Interaction Effects From Adjusted Models for Stage 1 (Self-Reported Hearing Difficulty) and Stage 5 (Obtaining a Hearing Aid)

Effect of SEP within age groups^a^	1. Self-reported hearing difficulty, *n* = 8,230	
Age group		
50–64	0.83***	0.78–0.89
65–74	0.85***	0.78–0.92
75+	0.98	0.88–1.08
LR test^b^	*χ* ^2^ = 7.09	*p* = .029
Effect of gender within age groups^c^	5. Hearing aid obtained, *n* = 818	
Age group		
50–64	2.60*	1.14–5.93
65–74	0.60	0.22–1.46
75+	2.07	0.95–4.50
LR test^b^	*χ* ^2^ = 6.43	*p* = .040

*Notes*. LR = likelihood ratio; SEP = socioeconomic position. All models adjusted for SEP, gender, age group, marital status, retirement status, and ownership of private health insurance.

^a^Odds ratio of progression in this stage of health-seeking behavior with an increase in one unit (z-score) of SEP in each age group from adjusted model including an interaction between SEP as a continuous variable and age group as a categorical variable.

^b^ *χ* ^2^ and *p* value for likelihood ratio test between adjusted model without interaction term and adjusted model with interaction term.

^c^Odds ratio of progression in this stage of health-seeking behavior for women compared with men in each age group from adjusted model including an interaction between gender as a binary variable and age group as a categorical variable.

*p* value for Wald test from adjusted model with interaction term, levels of significance: *p < .05. **p < .01. ***p < .001.

### Phase B: Diagnosis and Recommendation

In model 3, we did not observe an association between SEP and the odds of being referred to an ear specialist upon mentioning hearing difficulty to a health professional. There was strong evidence that people in the oldest age group had more than double the odds of being referred to an ear specialist. Among individuals who mentioned their hearing difficulty to a health professional (whether or not they were referred to an ear specialist), there was some evidence that an increase in SEP score was associated with a decrease in the odds of receiving a recommendation for a hearing aid (model 4). There was strong evidence that the odds of hearing aid being recommended increased with increasing age group. No interaction between age group and gender or age group and SEP was identified. Model 4 was further adjusted for referral to an ear specialist as a potential indicator capturing some of the variation related to the severity of hearing loss. This analysis showed that SEP was no longer associated with the odds of receiving a hearing aid recommendation (odds ratio = 0.89, 95% CI 0.77–1.02, *p* = .098). Additionally, the results revealed that individuals who were referred to an ear specialist had 19 times higher odds of receiving a hearing aid recommendation (95% CI 13.4–27.0, *p* < .001) compared with those who were not referred.

### Phase C: Hearing Aid Acquisition and Use

Analysis of progression in stage 5 showed no evidence of an association between SEP and obtaining a hearing aid among those who received a recommendation for one. However, the odds of hearing aid acquisition were strongly associated with age group, and further analysis showed that there was some evidence of interaction between age group and gender. The adjusted model including an interaction term between these variables showed that in the youngest age group (50–64) women had 2.6 times higher odds of acquiring a hearing aid compared with men (*p* < .05), whereas this effect was not seen in the older two age groups (Table 3). In the adjusted model predicting hearing aid use among hearing aid owners (stage 6), data were consistent with no association between SEP and reported use. Neither gender nor age group predicted hearing aid use, and no evidence of interaction was identified.

## Discussion

### Summary of Findings

The aim of this study was to examine the progression through health-seeking behaviors related to hearing loss among the older noninstitutionalized population in England. Our findings showed that the prevalence of hearing difficulty was 39.1% and less than 20% of those who reported perceiving a hearing handicap were using a hearing aid. Within the six stages from hearing difficulty to using a hearing aid, the largest proportion of potential respondents not progressing to the next stage of health-seeking behavior was in stage 2, where 54% of those with hearing difficulty had not approached a health professional.

Secondly, we assessed whether health-seeking behavior was associated with SEP. Previously described gradients in prevalence of self-reported hearing loss by age group and gender were confirmed (Smeeth et al., 2002). We found strong evidence that the odds of hearing loss were higher among those with lower SEP. This phenomenon may be related to higher lifetime exposure to noise among different occupational categories (Davis et al., 2007; Hasson, Theorell, Westerlund, & Canlon, 2010) and to a faster deterioration in overall physical health among older people from lower occupational grades (Chandola, Ferrie, Sacker, & Marmot, 2007). Data were consistent with no association between SEP and self-reported hearing difficulty among individuals older than 75 years, potentially a result of selective mortality and a strong influence of aging on the severity of hearing impairment in this age group. The data were consistent with no association of SEP with progression in the second stage of Phase A, where an individual, aware of their handicap, initiates contact with the health system. Men and respondents in the oldest age group were more likely to have mentioned their self-perceived hearing difficulty to a health professional.

Progression in Phase B takes place entirely within the health system. The data were consistent with no association between SEP and reporting having received a referral to an ear specialist. On the other hand, lower SEP carried marginally higher odds of a recommendation for a hearing aid. One possible explanation of this direction of inequality is higher severity of hearing loss among respondents with lower SEP, as shown in Step 1. Although the data set did not contain any objective measure of hearing loss, we used the referral to an ear specialist as a surrogate indicator of higher severity of hearing loss, as assessed by the health professional initially approached. Adjusting for ear specialist referral rendered the association between SEP and receipt of recommendation for hearing aid insignificant.

In Phase C, data were consistent with no association between SEP and hearing aid acquisition among respondents who reported receiving a recommendation or between SEP and hearing aid use among hearing aid owners. Hearing aids are available free of charge through the NHS. However, hearing aid users may encounter other direct and indirect expenditures related to treatment of hearing loss, such as transportation to fitting sessions and battery purchases. Although the range of hearing aid models available to those willing to pay privately is broader, our findings showed that these considerations did not act to create a significant gradient in access and utilization of treatment for hearing impairment. This finding contrasts with studies from other high-income countries, some of which do not provide universal access to health care and free hearing aids. Prospective rate of hearing aid acquisition in a U.S. study was strongly associated with educational level, a finding the authors partly attributed to higher income among individuals with higher levels of education (Fischer et al., 2011). In a sample of 65 years and older U.S. individuals who reported having received a recommendation for a hearing aid, income satisfaction (among women) and concerns about costs (among men) were significant predictors of hearing aid use (Garstecki & Erler, 1998). Our data do not provide insight into the reasons for nonuse of hearing aids among the 26.9% of our sample who own hearing aids. Even if we postulate that nonuse was due to lack of benefit, discomfort, or counseling efficacy, such determinants did not appear to be linked to individual SEP.

### Limitations

One of the limitations of this study is the self-reported nature of all six measures in the process of health-seeking behavior. The determinants of self-reporting hearing difficulty include the existence of this condition in sufficient severity to impede on an individual’s life, knowledge of this handicap (self-perception), and willingness to report its existence to the survey enumerator. Different types of bias may have occurred in self-reporting hearing loss, receiving a hearing aid recommendation and compliance with this recommendation. Because the objective severity of hearing impairment was not available, we could not adjust for it in our models. Although the sample analyzed is derived from a longitudinal cohort, detailed information about health-seeking behavior for hearing loss was available only in Wave 2. The cross-sectional nature of our analysis did not allow for information about the date and length of the health-seeking process (e.g., hearing loss diagnostic and hearing aid prescription protocols, waiting lists, and types of hearing aids available) to be considered.

However, we believe it is unlikely that the existence of hearing impairment could fully explain the existence of the gradient in self-reported hearing handicap, in light of research showing that there was no evidence that social mobility was caused by health status (Chandola, Bartley, Sacker, Jenkinson, & Marmot, 2003). The use of a latent variable to measure SEP did not allow for the effect of the separate four component variables to be disaggregated. This would need to be explored by using directed acyclic graphs in a suitable estimating framework such as path analysis, which is beyond the scope of this article. Lastly, our parameter estimates depend on the models being correctly specified. If important unknown unmeasured confounders were excluded from our analysis, the derived estimates may be biased.

## Conclusion and Recommendations

Among the older population in England, nearly half of self-perceived hearing difficulty is unreported to health professionals, and therefore remains untreated. SEP was strongly associated with odds of self-reported hearing difficulty. In light of a high prevalence of hearing difficulty and absence of a hearing loss screening program among older individuals in the United Kingdom, the strong associations between self-reported hearing difficulty and increasing age, male gender, and lower SEP may aid health practitioners in raising the issue with particular patients during other health care interactions. In regard to the objective of this study, we found that individuals of different SEP scores appeared to receive equitable levels of care upon approaching a health care professional with self-perceived hearing loss, and no SEP gradient was identified in ownership and utilization of hearing aids.

Women with a self-perceived hearing handicap were less likely to discuss its existence with a health professional, but more likely to obtain a hearing aid upon recommendation. Further research is needed to evaluate how women may be encouraged to report this sensory impairment and how uptake of hearing aids could be improved among men, especially in the youngest age group. Whereas the current system does not appear to disadvantage individuals from lower SEP backgrounds, any changes to screening or treatment availability and eligibility will need to assess the potential for creating such gradients.

In addition to the above recommendations targeted at clinical practice, we offer two potential avenues of future research to help elucidate the pathways linking SEP and health outcomes via health-seeking behaviors. Firstly, comparative country studies of accessibility and affordability of hearing loss care in various health system environments may assist in designing interventions to lower barriers to receiving appropriate treatment. Secondly, a more general exploration of the influence of health-seeking behavior in the causal framework between socioeconomic behavior and health outcomes is needed. A high proportion of hearing impairment is undiagnosed and untreated in the older population in England, and efforts need to be made to screen and encourage the use of hearing aids in order to prevent avoidable morbidity in this population. To guide effective policy design, future analysis needs to simultaneously explore the relative effects of all causal pathways (lifestyle factors, psychological aspects, health knowledge, environmental hazards, and health-seeking behaviors) linking SEP with health outcomes.

## Funding

This work was supported by the Economic and Social Research Council (ESRC) 1+3 Studentship grant award (ES/I903224/1) and a Medical Research Council (MRC) Population Health Science fellowship (G0802442).

## References

[CIT0001] Alpiner J. G. Roche V (1996). Hearing loss and tinnitus. In Jahnigen D. Schrier R (Eds.), Geriatric medicine (2nd ed., pp. 353–366). Cambridge, MA: Blackwell Science.

[CIT0002] Appollonio I. Carabellese C. Frattola L. Trabucchi M (1996). Effects of sensory aids on the quality of life and mortality of elderly people: A multivariate analysis. Age and Ageing, 25, 89–96. doi:10.1093/ageing/25.2.89 8670534 10.1093/ageing/25.2.89

[CIT0003] Bagai A. Thavendiranathan P. Detsky A. S (2006). Does this patient have hearing impairment? Journal of the American Medical Association, 295, 416–428. doi:10.1001/jama.295.4.416 16434632 10.1001/jama.295.4.416

[CIT0004] Banks J. Breeze E. Lessof C. Nazroo J (2006). Retirement, health and relationships of the older population in England The 2004 English Longitudinal Study of Ageing (Wave 2). London, UK: The Institute for Fiscal Studies.

[CIT0005] Braunack-Mayer A. Avery J (2009). Before the consultation: Why people do (or do not) go to the doctor. British Journal of General Practice, 59, 478–479. doi:10.3399/bjgp09X453495 19566994 10.3399/bjgp09X453495 PMC2702011

[CIT0006] Chandola T. Bartley M. Sacker A. Jenkinson C. Marmot M (2003). Health selection in the Whitehall II study, UK. Social Science and Medicine, 56, 2059–2072. doi:10.1016/S0277-9536(02)00201-0 12697197 10.1016/s0277-9536(02)00201-0

[CIT0007] Chandola T. Ferrie J. Sacker A. Marmot M (2007). Social inequalities in self reported health in early old age: Follow-up of prospective cohort study. BMJ, 334, 990. doi:10.1136/bmj.39167.439792.55 17468119 10.1136/bmj.39167.439792.55 PMC1867907

[CIT0008] Chang H. P. Ho C. Y. Chou P (2009). The factors associated with a self-perceived hearing handicap in elderly people with hearing impairment—results from a community-based study. Ear and Hearing, 30, 576–583. doi:10.1097/AUD.0b013e3181ac127a 19633566 10.1097/AUD.0b013e3181ac127a

[CIT0009] Cheshire H. Cox H. Lessof C. Taylor R (2006). Chapter 12: Methodology. In Banks J. Breeze E. Lessof C. Nazroo J (Eds.), English longitudinal study of ageing: Retirement, health and relationships of the older population in England: The 2004 English Longitudinal Study of Ageing (Wave 2) (pp. 367–383). London, UK: Institute of Fiscal Studies.

[CIT0010] Dalton D. S. Cruickshanks K. J. Klein B. E. Klein R. Wiley T. L. Nondahl D. M (2003). The impact of hearing loss on quality of life in older adults. The Gerontologist, 43, 661–668. doi:10.1093/geront/43.5.661 14570962 10.1093/geront/43.5.661

[CIT0011] Davies S. C (2012). Annual report of the Chief Medical Officer, Volume One, 2011, On the State of the Public’s Health. London, UK: Department of Health.

[CIT0012] Davis A. Smith P. Ferguson M. Stephens D. Gianopoulos I (2007). Acceptability, benefit and costs of early screening for hearing disability: A study of potential screening tests and models. Health Technology Assessment, 11, 1–274. doi:10.3310/hta11420 10.3310/hta11420 17927921

[CIT0013] Duijvestijn J. A. Anteunis L. J. Hoek C. J. Van Den Brink R. H. Chenault M. N. Manni J. J (2003). Help-seeking behaviour of hearing-impaired persons aged > or = 55 years; effect of complaints, significant others and hearing aid image. Acta Oto-laryngologica, 123, 846–850. doi: 10.1080/0001648031000719 14575400 10.1080/0001648031000719

[CIT0014] Fischer M. E. Cruickshanks K. J. Wiley T. L. Klein B. E. K. Klein R. Tweed T. S (2011). Determinants of hearing aid acquisition in older adults. American Journal of Public Health, 101, 1449–1455. doi:10.2105/AJPH.2010.300078 21680930 10.2105/AJPH.2010.300078 PMC3134488

[CIT0015] Galobardes B. Shaw M. Lawlor D. A. Lynch J. W. Davey Smith G (2006). Indicators of socioeconomic position (part 1). Journal of Epidemiology and Community Health, 60, 7–12. doi:10.1136/jech.2004.023531 16361448 10.1136/jech.2004.023531 PMC2465546

[CIT0016] Garstecki D. C. Erler S. F (1998). Hearing loss, control, and demographic factors influencing hearing aid use among older adults. Journal of Speech, Language & Hearing Research, 41, 527–537. doi:10.1044/jslhr.4103.527 10.1044/jslhr.4103.527 9638919

[CIT0017] Gopinath B. Schneider J. Hartley D. Teber E. McMahon C. M. Leeder S. R. Mitchell P (2011). Incidence and predictors of hearing aid use and ownership among older adults with hearing loss. Annals of Epidemiology, 21, 497–506. doi:10.1016/j.annepidem.2011.03.005 21514179 10.1016/j.annepidem.2011.03.005

[CIT0018] Gopinath B. Schneider J. Hickson L. McMahon C. M. Burlutsky G. Leeder S. R. Mitchell P (2012). Hearing handicap, rather than measured hearing impairment, predicts poorer quality of life over 10 years in older adults. Maturitas, 72, 146–151. doi:10.1016/j.maturitas.2012.03.010 22521684 10.1016/j.maturitas.2012.03.010

[CIT0019] Gopinath B. Schneider J. McMahon C. M. Teber E. Leeder S. R. Mitchell P (2012). Severity of age-related hearing loss is associated with impaired activities of daily living. Age and Ageing, 41, 195–200. doi:10.1093/ageing/afr155 22130560 10.1093/ageing/afr155

[CIT0020] Hasson D. Theorell T. Westerlund H. Canlon B (2010). Prevalence and characteristics of hearing problems in a working and non-working Swedish population. Journal of Epidemiology and Community Health, 64, 453–460. doi:10.1136/jech.2009.095430 19692714 10.1136/jech.2009.095430

[CIT0021] Heine C. Browning C. Cowlishaw S. Kendig H (2013). Trajectories of older adults’ hearing difficulties: Examining the influence of health behaviors and social activity over 10 years. Geriatrics and Gerontology International, 13, 911–918. doi:10.1111/ggi.12030 23311873 10.1111/ggi.12030

[CIT0022] Helvik A.-S. Wennberg S. Jacobsen G. Hallberg L (2008). Why do some individuals with objectively verified hearing loss reject hearing aids? Audiological Medicine, 6, 141–148. doi:10.1080/16513860802178692

[CIT0023] Hietanen A. Era P. Sorri M. Heikkinen E (2004). Changes in hearing in 80-year-old people: A 10-year follow-up study. International Journal of Audiology, 43, 126–135. doi:10.1080/14992020400050018 15198376 10.1080/14992020400050018

[CIT0024] Kiely K. M. Gopinath B. Mitchell P. Browning C. J. Anstey K. J (2012). Evaluating a dichotomized measure of self-reported hearing loss against gold standard audiometry: Prevalence estimates and age bias in a pooled national data set. Journal of Aging and Health, 24, 439–458. doi:10.1177/0898264311425088 22205434 10.1177/0898264311425088

[CIT0025] Knudsen L. V. Öberg M. Nielsen C. Naylor G. Kramer S. E (2010). Factors influencing help seeking, hearing aid uptake, hearing aid use and satisfaction with hearing aids: A review of the literature. Trends in Amplification, 14, 127–154. doi:10.1177/1084713810385712 21109549 10.1177/1084713810385712 PMC4111466

[CIT0026] Kramer S. E. Kapteyn T. S. Kuik D. J. Deeg D. J. H (2002). The association of hearing impairment and chronic diseases with psychosocial health status in older age. Journal of Aging and Health, 14, 122–137. doi:10.1177/089826430201400107 11892756 10.1177/089826430201400107

[CIT0027] Lin F. R. Yaffe K. Xia J. Xue Q. L. Harris T. B. Purchase-Helzner E., … Simonsick E. M (2013). Hearing loss and cognitive decline in older adults. JAMA Internal Medicine, 173, 293–299. doi:10.1001/jamainternmed.2013.1868 23337978 10.1001/jamainternmed.2013.1868 PMC3869227

[CIT0028] Lupsakko T. A. Kautiainen H. J. Sulkava R (2005). The non-use of hearing aids in people aged 75 years and over in the city of Kuopio in Finland. European Archives of Oto-Rhino-Laryngology, 262, 165–169. doi:10.1007/s00405-004-0789-x 15133689 10.1007/s00405-004-0789-x

[CIT0029] Mackenbach J. P. Stirbu I. Roskam A. R. Schaap M. M. Menvielle G. Leinsalu M. Kunst A. E (2008). Socioeconomic inequalities in health in 22 European countries. New England Journal of Medicine, 358, 2468–2481. doi:10.1056/NEJMsa0707519 18525043 10.1056/NEJMsa0707519

[CIT0030] Mackie A (2009). Adult hearing screening. London, UK: UK National Screening Committee, NHS.

[CIT0031] Maheswaran R. Pearson T. Jordan H. Black D (2006). Socioeconomic deprivation, travel distance, location of service, and uptake of breast cancer screening in North Derbyshire, UK. Journal of Epidemiology and Community Health, 60, 208–212. doi:10.1136/jech.200X.038398 16476749 10.1136/jech.200X.038398 PMC2465550

[CIT0032] Marmot M (2005). Social determinants of health inequalities. The Lancet, 365, 1099–1104. doi:10.1016/S0140-6736(05)71146-6 10.1016/S0140-6736(05)71146-6 15781105

[CIT0033] Meyer C. Hickson L. Khan A. Hartley D. Dillon H. Seymour J (2011). Investigation of the actions taken by adults who failed a telephone-based hearing screen. Ear and Hearing, 32, 720–731. doi:10.1097/AUD.0b013e318220d973 21697715 10.1097/AUD.0b013e318220d973

[CIT0034] Miles A. Rainbow S. Von Wagner C (2011). Cancer fatalism and poor self-rated health mediate the association between socioeconomic status and uptake of colorectal cancer screening in England. Cancer Epidemiology Biomarkers and Prevention, 20, 2132–2140. doi:10.1158/1055–9965.EPI-11–0453 10.1158/1055-9965.EPI-11-0453 PMC3199581 21953115

[CIT0035] Mondelli M. F. Souza P. J (2012). Quality of life in elderly adults before and after hearing aid fitting. Brazilian Journal of Otorhinolaryngology, 78, 49–56. doi:10.1590/S1808-86942012000300010 22714847 10.1590/S1808-86942012000300010 PMC9446233

[CIT0036] Murray C. J. L. Richards M. A. Newton J. N. Fenton K. A. Anderson H. R. Atkinson C., … Davis A (2013). UK health performance: Findings of the Global Burden of Disease Study 2010. The Lancet, 381, 997–1020. doi:10.1016/S0140-6736(13)60355-4 10.1016/S0140-6736(13)60355-4 23668584

[CIT0037] Pandhi N. Schumacher J. R. Barnett S. Smith M. A (2011). Hearing loss and older adults’ perceptions of access to care. Journal of Community Health, 36, 748–755. doi:10.1007/s10900-011-9369-3 21301940 10.1007/s10900-011-9369-3 PMC3197225

[CIT0038] Patel R. Lawlor D. A. Ebrahim S (2007). Socio-economic position and the use of preventive health care in older British women: A cross-sectional study using data from the British Women’s Heart and Health Study cohort. Family Practice, 24, 7–10. doi:10.1093/fampra/cml064 17158182 10.1093/fampra/cml064

[CIT0039] Ploubidis GB Benova L Grundy E Laydon D DeStavola B (2014). Lifelong socio economic position and biomarkers of later life health: Testing the contribution of competing hypotheses. Social Science & Medicine. doi: 10.1016/j.socscimed.2014.1002.1018 10.1016/j.socscimed.2014.02.018 24636422

[CIT0040] Ploubidis G. B. De Stavola B. L. Grundy E (2011). Health differentials in the older population of England: An empirical comparison of the materialist, lifestyle and psychosocial hypotheses. BMC Public Health, 11. doi:10.1186/1471-2458-1111-139 10.1186/1471-2458-11-390 PMC3128018 21612643

[CIT0041] Pronk M. Deeg D. J. Smits C. van Tilburg T. G. Kuik D. J. Festen J. M. Kramer S. E (2011). Prospective effects of hearing status on loneliness and depression in older persons: identification of subgroups. International Journal of Audiology, 50, 887–896. doi:10.3109/14992027.2011.599871 21929374 10.3109/14992027.2011.599871

[CIT0042] Ruan Q. Ma C. Zhang R. Yu Z (2013). Current status of auditory aging and anti-aging research. Geriatrics and Gerontology International, 14, 40–53. doi:10.1111/ggi.12124 23992133 10.1111/ggi.12124

[CIT0043] Salonen J. Johansson R. Karjalainen S. Vahlberg T. Isoaho R (2011). Relationship between self-reported hearing and measured hearing impairment in an elderly population in Finland. International Journal of Audiology, 50, 297–302. doi:10.3109/14992027.2010.549517 21303228 10.3109/14992027.2010.549517

[CIT0044] Saunders G. H. Chisolm T. H. Wallhagen M. I (2012). Older adults and hearing help-seeking behaviors. American Journal of Audiology, 21, 331–337. doi:10.1044/1059-0889(2012/12–0028) 23233518 10.1044/1059-0889(2012/12-0028)

[CIT0045] Sindhusake D. Mitchell P. Smith W. Golding M. Newall P. Hartley D. Rubin G (2001). Validation of self-reported hearing loss. The Blue Mountains Hearing Study. International Journal of Epidemiology, 30, 1371–1378. doi:10.1093/ije/30.6.1371 11821349 10.1093/ije/30.6.1371

[CIT0046] Smeeth L. Fletcher A. E. Ng E. Stirling S. Nunes M. Breeze E., … Tulloch A (2002). Reduced hearing, ownership, and use of hearing aids in elderly people in the UK—the MRC Trial of the Assessment and Management of Older People in the Community: A cross-sectional survey. The Lancet, 359, 1466–1470. doi:10.1016/S0140-6736(02)08433-7 10.1016/s0140-6736(02)08433-7 11988245

[CIT0047] Stevens G. Flaxman S. Brunskill E. Mascarenhas M. Mathers C. D. Finucane M (2011). Global and regional hearing impairment prevalence: An analysis of 42 studies in 29 countries. European Journal of Public Health, 23, 146–152. doi:10.1093/eurpub/ckr176 22197756 10.1093/eurpub/ckr176

[CIT0048] Stowasser T. Heiss F. McFadden D. Winter J (2011). “Health, wealthy and wise?” Revisited: An analysis of the causal pathways from socio-economic status to health *NBER Working paper series* (Working Paper 17273). Cambridge, MA: National Bureau of Economic Research.

[CIT0049] Taylor R. Conway L. Calderwood L. Lessof C (2002). Chapter 9: Methodology. In Marmot M. Banks J. Blundell R. Lessof C. Nazroo J (Eds.), Health, wealth and lifestyles of the older population in England: The 2002 English Longitudinal Study of Ageing (pp. 357–374). London, UK: Institute for Fiscal Studies.

[CIT0050] Valete-Rosalino C. M. Rozenfeld S (2005). Auditory screening in the elderly: Comparison between self-report and audiometry. Brazilian Journal of Otorhinolaryngology, 71, 193–200. doi:S0034-72992005000200013 16446917 10.1016/S1808-8694(15)31310-0 PMC9450545

[CIT0051] Wallhagen M. I (2010). The stigma of hearing loss. The Gerontologist, 50, 66–75. doi:10.1093/geront/gnp107 19592638 10.1093/geront/gnp107 PMC2904535

[CIT0052] Wallhagen M. I. Strawbridge W. J. Shema S. J. Kaplan G. A (2004). Impact of self-assessed hearing loss on a spouse: A longitudinal analysis of couples. Journal of Gerontology B Psychological Science and Social Science, 59, 190–196. doi:10.1093/geronb/59.3.S190 10.1093/geronb/59.3.s190 15118025

[CIT0053] Walters K. Iliffe S. Orrell M (2001). An exploration of help-seeking behaviour in older people with unmet needs. Family Practice, 18, 277–282. doi:10.1093/fampra/18.3.277 11356734 10.1093/fampra/18.3.277

[CIT0054] World Health Organization. (2008). The global burden of disease: 2004 update. Geneva, Switzerland: World Health Organization.

[CIT0055] Yamasoba T. Lin F. R. Someya S. Kashio A. Sakamoto T. Kondo K (2013). Current concepts in age-related hearing loss: Epidemiology and mechanistic pathways. Hearing Research, 303, 30–38. doi:10.1016/j.heares.2013.01.021 23422312 10.1016/j.heares.2013.01.021 PMC3723756

[CIT0056] Yueh B. Collins M. P. Souza P. E. Boyko E. J. Loovis C. F. Heagerty P. J., … Hedrick S. C (2010). Long-term effectiveness of screening for hearing loss: The screening for auditory impairment—which hearing assessment test (SAI-WHAT) randomized trial. Journal of the American Geriatrics Society, 58, 427–434. doi:10.1111/j.1532-5415.2010.02738.x 20398111 10.1111/j.1532-5415.2010.02738.x

